# What You Always Wanted to Know about Endovascular Therapy in Acute Ischemic Stroke but Never Dared to Ask: A Comprehensive Review

**DOI:** 10.31083/j.rcm2310340

**Published:** 2022-10-11

**Authors:** Philipp Bücke, Jose E. Cohen, Thomas Horvath, Alexandru Cimpoca, Pervinder Bhogal, Hansjörg Bäzner, Hans Henkes

**Affiliations:** ^1^Department of Neurology, Inselspital, Bern University Hospital, University of Bern, 3012 Bern, Switzerland; ^2^Department of Neurosurgery, Hadassah Medical Center, Hebrew University Jerusalem, 91905 Jerusalem, Israel; ^3^Neuroradiologische Klinik, Klinikum Stuttgart, 70174 Stuttgart, Germany; ^4^Interventional Neuroradiology Department, The Royal London Hospital, E1 1FR London, UK; ^5^Neurologische Klinik, Klinikum Stuttgart, 70174 Stuttgart, Germany; ^6^Medical Faculty, Universität Duisburg-Essen, 45141 Essen, Germany

**Keywords:** ischemic stroke, embolic stroke, embolectomy, endovascular procedure, acute stroke

## Abstract

In 2015, mechanical thrombectomy (MT) in combination with intravenous thrombolysis was demonstrated to be superior to best medical treatment alone in patients with anterior circulation stroke. This finding resulted in an unprecedented boost in endovascular stroke therapy, and MT became widely available. MT was initially approved for patients presenting with large vessel occlusion in the anterior circulation (intracranial internal carotid artery or proximal middle cerebral artery) within a 6-hour time window. Eventually, it was shown to be beneficial in a broader group of patients, including those without known symptom-onset, wake-up stroke, or patients with posterior circulation stroke. Technical developments and the implementation of novel thrombectomy devices further facilitated endovascular recanalization for acute ischemic stroke. However, some aspects remain controversial. Is MT suitable for medium or very distal vessel occlusions? Should emergency stenting be performed for symptomatic stenosis or recurrent occlusion? How should patients with large vessel occlusion without disabling symptoms be treated? Do certain patients benefit from MT without intravenous thrombolysis? In the era of personalized decision-making, some of these questions require an individualized approach based on comorbidities, imaging criteria, and the severity or duration of symptoms. Despite its successful development in the past decade, endovascular stroke therapy will remain a challenging and fascinating field in the years to come. This review aims to provide an overview of patient selection, and the indications for and execution of MT in patients with acute ischemic stroke.

## 1. Introduction 

Before the era of endovascular stroke therapy, intravenous thrombolysis (IVT) was the only approved therapeutic option for patients with acute ischemic stroke (within 4.5 hours of symptom onset) [1]. However, IVT is limited in its ability to dissolve emboli leading to intracranial large vessel occlusion (LVO) [2, 3]. In a considerable number of patients, symptoms do not improve sufficiently [4]. Compared with patients with stroke caused by other etiologies, patients with an LVO more frequently experience disability, dependency, or death [4, 5]. The longstanding conundrum of how to eradicate intracranial emboli has remained unanswered.

Endovascular stroke therapy initially focused on local intra-arterial administration of fibrinolytic agents (intra-arterial thrombolysis) [6]. Mechanical thrombectomy (MT) as a potential therapeutic strategy in patients with acute ischemic stroke and LVO was first described in 2001 [7]. The idea was to remove the clot with an intra-arterial catheter navigated to the site of the occlusion via a thrombus suction technique. Dedicated thrombectomy devices (e.g., the Merci Retriever [Concentric Medical, Mountain View, California, USA], the Penumbra system [Alameda, California, USA], and the phenox clot retriever [phenox GmbH, Bochum, Germany]) were developed and consecutively approved [8, 9, 10]. In 2013, a series of randomized controlled trials (RCTs), known as the “unhappy triad”, did not find a beneficial effect of MT over IVT in acute stroke treatment [11, 12, 13].

The Solitaire stent (Medtronic, Dublin, Ireland), a fully retrievable micro-catheter delivered stent, became a “game changer” for endovascular therapy in acute ischemic stroke [14]. After the initial development for the treatment of wide-necked cerebral aneurysms, it was observed that the stent could be pulled back without a need for stent closure [15], thus enabling successful retrieval of intracranial thrombi and complete recanalization of formerly occluded vessels. The demonstration that stent retriever MT was superior to first-generation devices [16] led to an unprecedented boost in neuro-endovascular therapy, thus promoting the development of subsequent thrombectomy devices (e.g., the Trevo retriever [Stryker, Kalamazoo, Michigan, USA]) [17]. Eventually, five RCTs demonstrated the superiority of MT plus IVT to IVT alone in patients with an occlusion of the middle cerebral artery (MCA; M1 segment) or the intracranial internal carotid artery (ICA) [18, 19, 20, 21, 22].

With the continual expansion of indications, more patients can benefit from MT, including patients with wake-up stroke or unknown symptom onset, in an advanced time-window beyond 6 hours of symptom onset, or with more distal occlusions (e.g., proximal M2 segment of the MCA) [23, 24, 25]. Novel technical developments, such as the direct aspiration first-pass technique (ADAPT) or a combined stent retriever and distal aspiration approach, might lead to further improvements in efficacy and safety [26, 27]. Despite this progress, a striking number of uncertainties remain. What about medium or distal vessel occlusions? Should MT in posterior circulation stroke be performed on a regular basis? Is an emergency stenting procedure necessary in cases of symptomatic stenosis or recurrent occlusions? How should patients with LVO without disabling symptoms be treated? Do certain patients benefit from MT without intravenous thrombolysis?

In this review, we aim to provide an overview of MT indications, patient selection, technical aspects, and potential complications. The current evidence regarding the remaining controversies will be discussed. We aim to present treatment strategies with a focus on borderline decision-making and potential future developments.

## 2. Discussion

### 2.1 Patients

#### 2.1.1 Patient Selection 

LVO is an occlusion of the most proximal intracranial vessels, such as the ICA, the M1 or the proximal M2 segments of the MCA, the A1 segment of the anterior cerebral artery (ACA), the BA, the vertebral artery, or the P1 segment of the posterior cerebral artery (PCA). More distal occlusions (e.g., distal M2, M3, A2, and P2) can be classified as medium vessel occlusions. However, inconsistencies exist as some authors categorize M2, A1, and P1 as medium vessel occlusions [28].

**Fig. 1.**
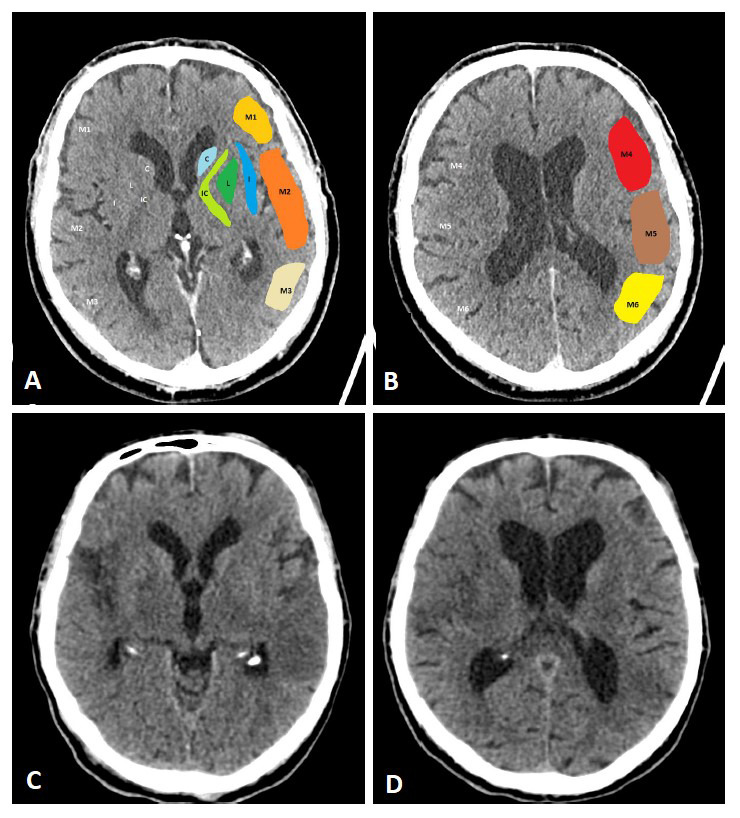
**ASPECTS**. Visualization of the ASPECTS territories (A,B). The following areas are covered: M1 (anterior MCA cortex, frontal operculum), M2 (anterior temporal lobe, laterally to the insula), M3 (posterior temporal lobe, posterior MCA cortex), M4 (anterior MCA cortex superior to M1), M5 (lateral MCA cortex superior to M2), M6 (posterior MCA cortex superior to M3), insula (I), internal capsule (IC), caudate (C), and lentiforme nucleus (L). Each area accounts for 1 point. The maximum ASPECTS score is 10. Hypodensity in a described area leads to a deduction of one point. (C,D) show an example of CT ASPECTS. Hypodensity in the M2 and M6 areas is observed. Total ASPECTS: 8.

Within the 6-hour time window, the selection of patients with anterior circulation stroke eligible for MT is based on non-contrast computed tomography (NCCT) or magnetic resonance imaging (MRI), including angiography (CT-A or MR-A). The Alberta Stroke Program Early CT Score (ASPECTS, based on NCCT) estimates the amount of infarcted brain parenchyma in the MCA territory (Fig. 1) [29]. Overall, a beneficial effect of MT can be expected in patients with ASPECTS ≥6 [30]. MRI can rule out intracranial hemorrhage as reliably as NCCT using gradient recalled echo sequences (GRE) [31]. Diffusion-weighted imaging (DWI) is used to visualize the infarct core. In patients with acute stroke, DWI-ASPECTS is an average of 1 point lower than ASPECTS based on NCCT [32]. A DWI-ASPECTS ≥5 is associated with good functional outcomes after MT [33].

**Fig. 2.**
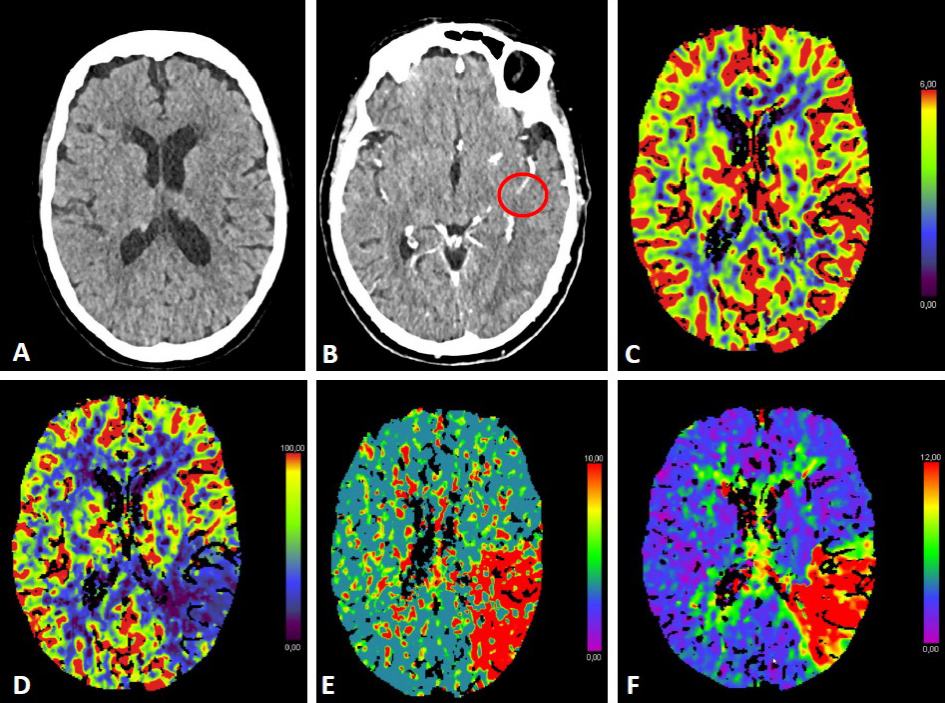
**CT-Perfusion**. NCCT ASPECTS 10 (A). CT-A with M2-occlusion (B). Interpretation of CT-P: the cerebral blood volume (CBV) is symmetrical without a regional decrease (C). Cerebral blood flow (CBF) is reduced in the posterior MCA territory on the left (D). The mean transit time (MTT) of the contrast agent (E) and Tmax (time to maximum; time delay between the contrast agent arrival in the proximal large vessel arterial circulation and the brain parenchyma perfusion [F]) are prolonged. The infarct core in CT-P shows a markedly reduced CBF (<25 mL/100 g/min) and CBV (<2 mL/100 g) together with an increase in MTT and Tmax [38, 39]. Penumbral tissue shows a delay in MTT (>145%) and Tmax (>6 sec) as well as a reduced CBF and a normal or slightly increased CBV. Beyond these absolute values, relative CT-P thresholds are mentioned (e.g., infarct core [CBF] defined as <30% of the contralateral CBF) [40]. CT-P parameters in Fig. 2 suggest a large penumbra with prolonged MTT and Tmax. There is a reduction in CBF with normal CBV. MT was performed in this patient.

The use of additional perfusion imaging (CT-P or MR-P) in the early time window (within 6 hours of symptom onset) is controversial and is not recommended in routine clinical practice [34, 35, 36, 37]. Perfusion imaging can be used to estimate the infarct core and potential tissue at risk (penumbra). Fig. 2 (Ref. [38, 39, 40]) illustrates CT-P parameters and potential thresholds for both the infarct core and penumbra. These thresholds are debatable and not universally accepted, and may change with the duration of symptoms [38, 40]. In an early time-window, CT-P can overestimate the infarct core, possibly because of a lack of contrast arrival overall [41]. In a pooled analysis from the Highly Effective Reperfusion evaluated in Multiple Endovascular Stroke Trials (HERMES) collaboration, adding CT-P in an early time window has not been found to be associated with functional outcomes [37].

**Fig. 3.**
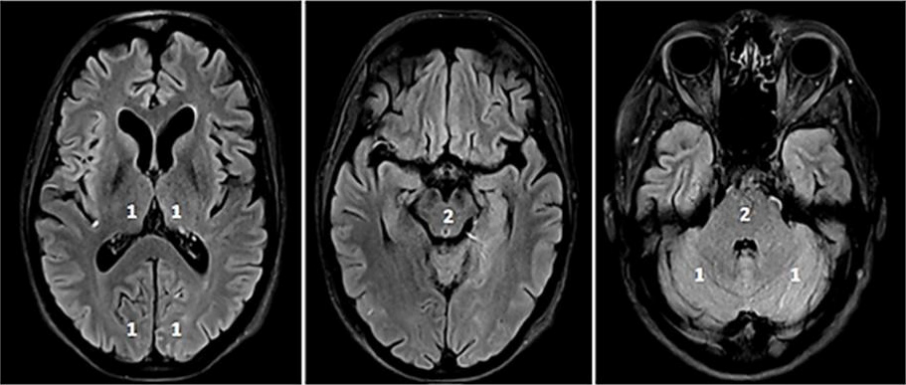
**pc-ASPECTS (posterior circulation ASPECTS)**. The pc-ASPECTS is a 10-point score evaluating the extent of ischemia in the posterior circulation. Scores of 10 points indicate no signs of ischemia in NCCT or MRI (diffusion weighted imaging, DWI). Each thalamus, occipital lobe and cerebellar hemisphere accounts for 1 point, and the mesencephalon and pons account for 2 points. Fig. 3 shows fluid attenuated inversion recovery (FLAIR) sequences because of better image quality.

In posterior circulation stroke, MT is currently recommended in carefully selected patients with BA occlusion [34, 35, 36]. Imaging tools such as the posterior circulation collateral score (PC-CS) or posterior-circulation ASPECTS (pc-ASPECTS; Fig. 3) might contribute to the decision-making process [42]. A pc-ASPECTS ≥5 appears to be a reasonable cut-off even in late-presenting patients [43].

Current guideline recommendations for patient selection and treatment indications for MT (notably the European Stroke Organisation [ESO]–European Society for Minimally Invasive Neurological Therapy [ESMINT], American Heart Association [AHA]/American Stroke Association [ASA], and Chinese Stroke Association [CSA]) are summarized in Table 1 [34, 35, 36].

**Table 1.** **Summary of guideline recommendations for endovascular stroke therapy [34, 35, 36]**.

European Stroke Organisation (ESO) - European Society for Minimally Invasive Neurological Therapy (ESMINT): Guidelines on Mechanical Thrombectomy in Acute Ischemic Stroke	*Patient selection*: In adults with anterior circulation large vessel occlusion-related acute ischemic stroke presenting within 6 hours after symptom onset, we recommend mechanical thrombectomy plus best medical management—including intravenous thrombolysis whenever indicated—over best medical management alone to improve functional outcome.
	*Unknown symptom onset*: In adults with anterior circulation large vessel occlusion-related acute ischemic stroke presenting between 6 and 24 hours from time last known well and fulfilling the selection criteria of DEFUSE-3 or DAWN, we recommend mechanical thrombectomy plus best medical management over best medical management alone to improve functional outcome.
	*Bridging therapy*: In patients with large vessel occlusion-related ischemic stroke eligible for both treatments, we recommend intravenous thrombolysis plus mechanical thrombectomy over mechanical thrombectomy alone. Both treatments should be performed as early as possible after hospital arrival. Mechanical thrombectomy should not prevent the initiation of intravenous thrombolysis, and intravenous thrombolysis should not delay mechanical thrombectomy.
	*Imaging*: In adult patients with anterior circulation large vessel occlusion-related acute ischemic stroke presenting from 0 to 6 hours from time last known well, advanced imaging is not necessary for patient selection.
	*Age*: We recommend that patients aged ≥80 years with large vessel occlusion-related acute ischemic stroke within 6 hours of symptom onset should be treated with mechanical thrombectomy plus best medical management, including intravenous thrombolysis whenever indicated. Application of an upper age limit for mechanical thrombectomy is not justified.
Guidelines for the Early Management of Patients With Acute Ischemic Stroke: 2019 Update to the 2018 Guidelines for the Early Management of Acute Ischemic Stroke: A Guideline for Healthcare Professionals From the American Heart Association/American Stroke Association	*Patient selection*: Patients eligible for IV alteplase should receive IV alteplase even if mechanical thrombectomy is being considered.
	*Patient selection*: Patients should receive mechanical thrombectomy with a stent retriever if they meet all the following criteria: (1) prestroke mRS score of 0 to 1; (2) causative occlusion of the internal carotid artery or MCA segment 1 (M1); (3) age ≥18 years; (4) NIHSS score of ≥6; (5) ASPECTS of ≥6; and (6) treatment can be initiated (groin puncture) within 6 hours of symptom onset.
	*Patient selection*: Although the benefits are uncertain, the use of mechanical thrombectomy with stent retrievers may be reasonable for carefully selected patients with AIS in whom treatment can be initiated (groin puncture) within 6 hours of symptom onset and who have causative occlusion of the MCA segment 2 (M2) or MCA segment 3 (M3) portion of the MCAs.
	*Patient selection*: Although the benefits are uncertain, the use of mechanical thrombectomy with stent retrievers may be reasonable for carefully selected patients with AIS in whom treatment can be initiated (groin puncture) within 6 hours of symptom onset and who have causative occlusion of the anterior cerebral arteries, vertebral arteries, basilar artery, or posterior cerebral arteries.
	*Unknown symptom onset*: When selecting patients with AIS within 6 to 24 hours of last known normal who have LVO in the anterior circulation, obtaining CT-P or DW-MRI, with or without MRI perfusion, is recommended to aid in patient selection for mechanical thrombectomy, but only when patients meet other eligibility criteria from one of the RCTs that showed benefit from mechanical thrombectomy in this extended time window.
	*Unknown symptom onset*: In selected patients with AIS within 6 to 16 hours of last known normal who have LVO in the anterior circulation and meet other DAWN or DEFUSE 3 eligibility criteria, mechanical thrombectomy is recommended.
	*Imaging*: When evaluating patients with AIS within 6 hours of last known normal with LVO and an Alberta Stroke Program Early Computed Tomography Score (ASPECTS) of ≥6, selection for mechanical thrombectomy based on CT and CTA or MRI and MRA is recommended in preference to performance of additional imaging such as perfusion studies.
	*Technique*: Direct aspiration thrombectomy as first-pass mechanical thrombectomy is recommended as noninferior to stent retriever for patients who meet all the following criteria: (1) prestroke mRS score of 0 to 1; (2) causative occlusion of the internal carotid artery or M1; (3) age ≥18 years; (4) NIHSS score of ≥6; (5) ASPECTS ≥6; and (6) treatment initiation (groin puncture) within 6 hours of symptom onset.
	*Technique*: The use of a proximal balloon guide catheter or a large-bore distal-access catheter, rather than a cervical guide catheter alone, in conjunction with stent retrievers may be beneficial.
	*Tandem occlusions*: Treatment of tandem occlusions (both extracranial and intracranial occlusions) when performing mechanical thrombectomy may be reasonable.
Chinese Stroke Association guidelines for clinical management of cerebrovascular disorders: executive summary and 2019 update of clinical management of ischemic cerebrovascular diseases	*Patient selection*: Mechanical thrombectomy is strongly recommended for patients within 6 hours after AIS if they meet all the following criteria: (1) prestroke mRS score of 0–1; (2) causative occlusion of the internal carotid artery (ICA) or middle cerebral artery (MCA) segment 1 (M1); (3) age ≥18 years; (4) NIHSS score of ≥6 and (5) ASPECTS of ≥6.
	*Patient selection*: Mechanical thrombectomy with stent retrievers may be reasonable for carefully selected patients with AIS in whom treatment can be initiated (groin puncture) within 6 hours of symptom onset and who have causative occlusion of the MCA segment 2 (M2) or MCA segment 3 (M3) portion of the MCAs. Mechanical thrombectomy with stent retrievers may be reasonable for carefully selected patients with AIS in whom treatment can be initiated (groin puncture) within 6 hours of symptom onset and who have causative occlusion of the anterior cerebral arteries, vertebral arteries, basilar artery or posterior cerebral arteries.
	*Unknown symptom onset*: If feasible, patients with AIS within 6–24 hours of last known normal who have large vessel occlusion (LVO) in the anterior circulation, obtaining CT perfusion (CT-P) or diffusion‐weighted imaging (DWI) with MRI perfusion is recommended to aid in patient selection for endovascular therapy. Patient selected for endovascular therapy should follow the same eligibility criteria of the two major RCTs (DWI or CT-P Assessment With Clinical Mismatch in the Triage of Wake-Up and Late Presenting Strokes Undergoing Neurointervention With Trevo (DAWN) and Endovascular Therapy Following Imaging Evaluation for Ischemic Stroke 3 (DEFUSE 3).
	*Unknown symptom onset*: In selected patients with AIS within 6–16 hours of last known normal who have LVO in the anterior circulation and meet other DAWN or DEFUSE 3 eligibility criteria, mechanical thrombectomy is recommended.
	*Imaging*: It is unclear whether using perfusion imaging (CTP or perfusion weighted imaging) for selecting patients for endovascular treatment <6 hours is beneficial.
	*Bridging therapy*: Endovascular treatment should be performed as soon as possible after its indication. Patients eligible for IV rt-PA should receive IV rt-PA and direct perform bridging treatment for mechanical thrombectomy.

IV, intravenous; MCA, middle cerebral artery; mRS, modified Rankin scale; NIHSS, National Institutes of Health Stroke Scale; ASPECTS, Alberta Stroke Program Early CT Score; LVO, large vessel occlusion; CT-P, CT-Perfusion; AIS, acute ischemic stroke; rt-PA, recombinant tissue plasminogen activator.

#### 2.1.2 Patients with Unknown Symptom Onset 

After the publication of the DWI or CTP Assessment With Clinical Mismatch in the Triage of Wake-Up and Late Presenting Stroke Undergoing Neurointervention With TREVO (DAWN) and Endovascular Therapy Following Imaging Evaluation for Ischemic Stroke 3 (DEFUSE-III) trials in 2018, the indications for MT for anterior-circulation stroke (ICA or MCA [M1, M2]) were expanded to patients arriving up to 24 hours after symptom onset or in an unknown time window [23, 24]. The DAWN protocol required CT-P (with a CBF-threshold) or MRI (DWI) with a subsequent predefined age-dependent clinical-core mismatch (A: >80 years: NIHSS >10, core volume <21 mL; B: <80 years, NIHSS >10, core volume <31 mL; C: < 80 years, NIHSS >20, core volume 31–51 mL) [24]. DEFUSE-III required a perfusion-core mismatch on CT-P or MR-P, defined as an infarct core <70 mL, a penumbra >15 mL (T-max delay >6 seconds), and a penumbra/core ratio >1.8 [23]. Both RCTs demonstrated the superiority of MT to standard care in eligible patients. The findings translated well into daily practice: large retrospective studies found MT to be effective and safe even with the application of less strict selection criteria [44, 45, 46, 47].

Multimodal imaging modalities such as CT-P or MRI might not be available at all times. Thus, focusing on those modalities alone could withhold a potentially beneficial therapy from patients. Hendrix *et al*. [48] have reported similar outcomes in patients in early and late time windows who were treated after NCCT and CT-A (criteria: ASPECTS >6; retrospective analysis). This finding has been supported by data suggesting that CT ASPECTS and CT-P-parameters were similar in predicting DWI lesions in patients with acute ischemic stroke [49]. In the CT for Late Endovascular Reperfusion (CLEAR) cohort, patients presenting 6 hours after symptom onset selected for therapy after NCCT/CT-A versus CT-P or MRI did not differ in outcomes [50]. The results from a post-hoc-analysis of DEFUSE-III data have suggested that an ASPECTS of 8–10 is associated with better functional outcomes [51]. The MT treatment effects remained stable regardless of baseline ASPECTS or infarct core volume (CT-P). This finding might suggest that patients with a mismatch in perfusion imaging (and meeting the DEFUSE-III inclusion criteria) could benefit from MT regardless of ASPECTS and infarct core volume [51]. However, because of the retrospective design and consecutively introduced biases, these results must be interpreted cautiously and require future validation.

For BA occlusions, preliminary data of The Basilar Artery Chinese Endovascular Trial (BAOCHE) have been presented at the European Stroke Organisation Conference (ESOC) 2022 in Lyon, France [52]. Patients with a BA (or bilateral V4) occlusion within 6 to 24 hours of symptom onset/last-seen-well (ineligible for IVT or IVT with futile recanalization), NIHSS 6 or higher, pc ASPECTS <6 and a pons-midbrain index of two or lower were eligible. Functional outcome (mRS 0–3) was significantly higher in the MT group (46.4%; compared to 24.3%; OR 2.92 [1.56–5.47]).

### 2.2 Bridging Therapy 

#### 2.2.1 MT after IVT

Several studies have found MT plus standard care (IVT; bridging therapy) to be superior to IVT alone [18, 19, 20, 21, 22]. Most patients in the intervention arm (between 68% and 100%) were treated with IVT before MT. Subsequently, the question arose as to whether direct MT (without IVT) might lead to comparable results, thus sparing patients from potentially harmful IVT complications, such as intracerebral hemorrhage (ICH). In a HERMES collaboration analysis, MT has been found to be beneficial independently of IVT use [53].

In 2020 and 2021, three RCTs comparing direct MT and bridging therapy presented inconclusive results [54, 55, 56]. Different non-inferiority margins (NIMs) were defined in each study. In DIRECT-MT (NIM 0.8 [meaning that the lower boundary of the 95% confidence interval was 0.8 or higher]; odds ratio (OR) 1.07 [95% CI 0.81–1.40]), and Direct Endovascular Thrombectomy versus Combined IVT and Endovascular Thrombectomy for Patients With Acute Large Vessel Occlusion in the Anterior Circulation (DEVT; NIM –10% of the proportion of functional independent patients; –7.7%; OR 1.48 [0.81–2.74]) direct MT was non-inferior. In the Direct Mechanical Thrombectomy in Acute LVO Stroke (SKIP) study (NIM 0.74; 1.09 [97.5% CI 0.63 to ∞]), non-inferiority was not demonstrated. Wide confidential intervals, the dosage of IVT (0.6 mg/kg versus 0.9 mg/kg), and an entirely Asian patient population limited generalizability. Subsequent meta-analyses including observational data provided inconclusive results regarding outcomes and treatment complications [57, 58, 59, 60, 61, 62]. However, in the case of multiple passages, IVT appears to be associated with less disability and a smaller overall stroke volume [63]. The Multicenter Randomized Clinical trial of Endovascular treatment for Acute ischemic stroke in the Netherlands (MR CLEAN)-NO IV study showed neither non-inferiority (NIM 0.8; 0.84 [0.62–1.15]) nor superiority of direct MT [64]. In 2021, preliminary results of the Solitaire™ With the Intention For Thrombectomy Plus Intravenous t-PA Versus DIRECT Solitaire™ Stent-retriever Thrombectomy in Acute Anterior Circulation Stroke (SWIFT-DIRECT) and A Randomized Controlled Trial of DIRECT Endovascular Clot Retrieval versus Standard Bridging Therapy (DIRECT-SAFE) trials were presented at the ESOC and the World Stroke Congress [65, 66]. Neither trial confirmed non-inferiority. To identify specific subgroups of patients who might benefit from one of the two therapeutic options, independent patient data meta-analyses are currently in preparation. Beyond an overall interpretation of these results, a debate is necessary regarding clinically acceptable overall non-inferiority margins. Other developments such as the effect of the neuroprotectant nerinetide (ESCPAE-NEXT; NCT04462536) in direct MT, or the use of tenecteplase rather than alteplase for bridging therapy, might further influence future decision-making [67, 68]. Current guidelines (e.g., the 2022 ESO/ESMINT recommendations on IVT before MT) do not suggest skipping IVT in eligible patients [69].

Patients in need of secondary transfer have not been included in these analyses. Longer transportation times lead to later recanalization. With the “drip-and-ship” concept, eligible patients receive IVT treatment during transfer for MT. Bridging therapy in this context is effective and safe, and has been found to be a strong independent predictor of early recanalization and favorable outcomes [70, 71, 72]. A potential fragmentation or distal translocation of the thrombus caused by IVT appears to be associated with better functional outcomes, possibly because of smaller final infarct size [73].

No RCT has investigated bridging therapy in BA occlusion. However, a recent cohort study has demonstrated the superiority of the bridging concept [74]. The findings must be confirmed in future trials.

#### 2.2.2 Early Recanalization after IVT 

Early recanalization in embolic LVO can be spontaneous or an effect of IVT. Analyses in the Endovascular treatment for Small Core and Anterior circulation Proximal occlusion with Emphasis on minimizing CT to recanalization times (ESCAPE) trial have indicated that early recanalization (demonstrated in an 8-hour follow-up CT-A) is crucial for functional outcomes [75]. Recanalization occurs in approximately 40% of patients treated with IVT. IVT recanalization has been found to depend on the clot length, collaterals and localization: 4.4% ICA, 32.3% M1, 30.8% M2 and 4% of BA occlusions [3]. In the case of bridging therapy, up to 10% of patients recanalized as detected in digital subtraction angiography [76]. In medium-size vessel occlusion, only 50% of patients experience early recanalization. This result translates into outcomes: only every second patient achieves an excellent outcome, defined by a modified Rankin scale (mRS) score of 0–1 [77]. If IVT leads to early recanalization, the outcome is favorable. However, the overall recanalization rates are lower than those with (additional) MT [18, 19, 20, 21, 22].

### 2.3 Conscious Sedation and General Anesthesia

Whether conscious sedation (CS) is superior to general anesthesia (GA) in MT is a longstanding and ongoing debate. Retrospective and observational studies have reported contradictory results. Some studies have found that GA and CS are similar, whereas others have demonstrated superiority of either of the two methods [78, 79, 80, 81, 82]. A pooled analysis from the HERMES collaboration has indicated the superiority of CS over GA in terms of patient outcomes [83]. Because of the retrospective nature of the analysis, information on why GA was chosen over CS or vice versa is lacking. Clinical conditions such as severe coma or agitation due to aphasia often require GA, thus introducing considerable bias [83, 84].

RCTs conducted in Europe, China, and the US have not found either CS or GA to be superior to the other (see Table 2, Ref. [85, 86, 87, 88]). These studies have been limited by small sample sizes and single-center designs preventing generalizability of the findings (because the results may vary depending on local protocols, the experience of the treatment team, the choice of anesthetic drugs, and thresholds for vital signs such as blood pressure [BP]). Subsequent meta-analyses of individual patient data have found GA to be superior in terms of functional independence and recanalization rates [89, 90]. These results require cautious interpretation, because of the aforementioned limitations in the included RCTs. Multi-center trials with larger sample sizes are ongoing (e.g., SEGA [NCT 03263117]) [91].

**Table 2.** **General anesthesia versus conscious sedation**.

	SIESTA [85]	AnStroke [86]	GOLIATH [87]	Ren *et al*. [88]
*Year*	2016	2016	2018	2020
*Country*	Germany	Sweden	Denmark	China
*Sample size*	GA: 73; CS 77	GA 45; CS: 45	GA: 65; CS: 63	GA: 48; CS: 42
*Primary endpoint*	NIHSS at 24 h (improvement)	mRS at 3 months	Infarct growth (48–72 h)	mRS at 3 months
*Functional independence (mRS 0–2)*				
GA versus CS (n [%])	27 (37); 14 (18.2)	19 (42.2); 18 (40)	2 (1–3); 2 (1–4)*	2.5 (2–3); 2.5 (2–3)*
OR (CI); *p*-value	diff.: –18.8 (–32.8 to –4.8); 0.01	1	0.04	0.65
*Successful recanalization (mTICI 2b/3)*				
GA versus CS (n, %)	65 (89); 62 (80.5)	41 (91.1); 40 (88.9)	50 (76.9); 38 (60.3)	36 (85.7); 42 (87.5)
OR (CI); *p*-value	diff.: –8.5 (–19.9 to –2.9); 0.68	1	0.04	1
*NIHSS after 24 h (after 48 h in [85])*				
GA versus CS (mean [SD])	13.6 (11.1); 13.6 (9)	8 (3–15); 9 (2–15)*	6 (3–14); 10 (12–19)*	9 (7–11.25); 9 (7–11)*
OR (CI); *p*-value	diff.: 0.0 (–3.3 to –3.3); >0.99	0.59	0.19	0.49

* median (interquartile range) SIESTA, Sedation versus Intubation for Endovascular Stroke Treatment; AnStroke, Anesthesia During Stroke; GOLIATH, General or Local Anesthesia in Intra Arterial Therapy; GA, general anesthesia; CS, conscious sedation; mRS, modified Rankin scale; n, number; OR, Odds ratio; CI, confidence interval; NIHSS, National Institutes of Health Stroke Scale; SD, standard deviation; diff., difference.

Elevated BP during MT appears to be associated with favorable outcomes. Whereas some researchers have suggested a systolic BP above 140 mmHg, others aim for higher values (e.g., >20% higher than baseline BP) [92, 93]. A medication-induced systolic BP decrease in patients undergoing GA is frequently observed after initiation. As shown by Fandler-Höfler and colleagues, a single decrease in the mean BP below 60 mmHg might be associated with poorer outcomes [94]. BP goals and potential (not documented) BP drops might explain some of the inconsistencies in the available data. After all, “the conduct rather than the method of anesthesia” might determine outcomes [95]. No general recommendations exist regarding which method to use. Instead individual decisions need to be made according to the infrastructure, expertise, and local protocols.

### 2.4 Technical Aspects

#### 2.4.1 First-Pass Effect

**Fig. 4.**
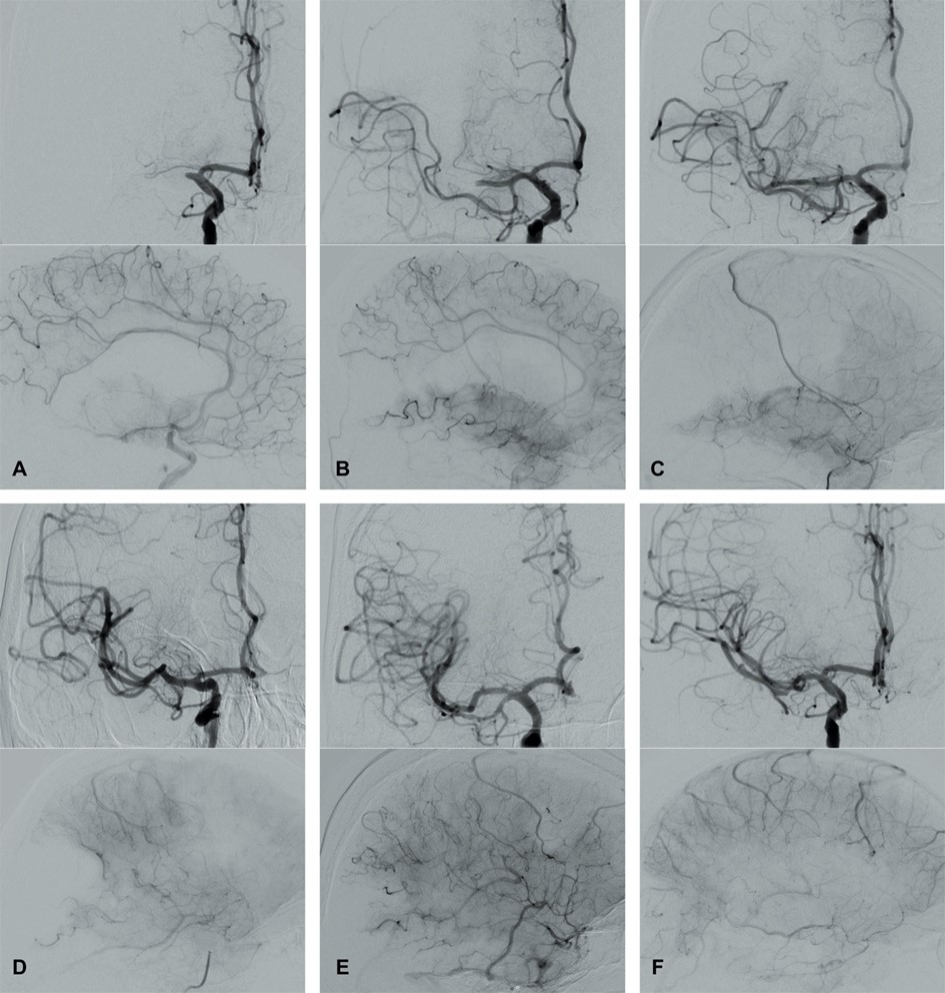
**Modified Thrombolysis in Cerebral Infarction (mTICI) grading system for evaluating the therapeutic success of IVT**. No perfusion of the right MCA - mTICI 0 (A). Antegrade reperfusion past the initial occlusion with only filling of a temporal branch of the right MCA - mTICI 1 (B). Antegrade reperfusion of only the superior division of the right MCA - mTICI 2a (C). Antegrade reperfusion of more than half of the previously occluded right MCA territory with persistent filling defect parieto-occipital - mTICI 2b (D). Near complete perfusion except for some distal emboli in several distal cortical vessels frontal and occipital - mTICI 2c (E). Complete antegrade reperfusion of the previously occluded right MCA - mTICI 3 (F).

In 2003, Higashida *et al*. [96] developed the Thrombolysis in Cerebral Infarction (TICI) grading system for evaluating the therapeutic success of IVT (Fig. 4 and Table 3, Ref. [96, 97, 98, 99]). The TICI score is derived from the Thrombolysis in Myocardial Infarction (TIMI) risk score. TICI scores of 0 or 1 indicate no or limited perfusion, respectively. TICI scores of 2a and 2b describe anterograde reperfusion of less or more than half of the occluded target artery previously ischemic territory, respectively. A TICI score of 3 indicates complete reperfusion without any visible distal vessel occlusion. The TICI system has been adapted and modified (mTICI, which is commonly used in both the literature and routine clinical practice) to include an additional TICI 2c category indicating near-complete perfusion except for slow flow or distal emboli in several distal cortical vessels [97, 98]. An excellent reperfusion outcome is defined by mTICI scores of 2c/3. The HERMES collaborators described the expanded TICI (eTICI) score in 2019 [99]. MRS-shift analyses at 90 days after MT have suggested differences in outcomes depending on the percentage of recanalized brain tissue (Table 3). The eTICI has been found to be an independent predictor of outcomes.

**Table 3.** **Thrombolysis in Cerebral Infarction (TICI) grading system [96, 97, 98, 99]**.

*grade*	TICI	mTICI	eTICI	*grade*
0	no perfusion	no perfusion	no perfusion	0
1	penetration with minimal perfusion	antegrade reperfusion past the initial occlusion, but limited distal branch filling with little or slow distal reperfusion	reduction in thrombus but without any resultant filling of distal arterial branches	1
2a	only partial filling (less than two-thirds) of the entire vascular territory is visualized	antegrade reperfusion of less than half of the occluded target artery previously ischemic territory (e.g., in one major division of the middle cerebral artery (MCA) and its territory)	reperfusion of 1–49% of the territory	2a
2b	complete filling of all of the expected vascular territory is visualized but the filling is slower than normal	antegrade reperfusion of more than half of the previously occluded target artery ischemic territory (e.g., in two major divisions of the MCA and their territories)	reperfusion of 50–66% of the territory	2b50
			reperfusion of 67–89% of the territory	2b67
2c	n.a.	near complete perfusion except for slow flow or distal emboli in a few distal cortical vessels	extensive reperfusion of 90–99% of the territory	2c
3	complete perfusion	complete antegrade reperfusion of the previously occluded target artery ischemic territory, with absence of visualized occlusion in all distal branches	complete or full reperfusion (100% reperfusion)	3

MCA, middle cerebral artery; n.a., not applicable; mTICI, modified Thrombolysis in Cerebral Infarction score; eTICI, expanded Thrombolysis in Cerebral Infarction Score.

First-pass reperfusion (FPR) describes the effect of excellent reperfusion following the first thrombectomy device pass (i.e., the first attempt to re-open the vessel). It was initially described with the Solitaire stent retriever in anterior circulation stroke. Many studies have found an association of FPR with outcomes [100, 101]. The time taken for each additional pass, and consequent continued growth of the infarction, makes an excellent functional outcome less likely, even in the case that excellent reperfusion is finally achieved [102, 103, 104]. In retrospective analyses, factors such as the site of the vessel occlusion (M1), door to groin time, and baseline ASPECTS have been found to influence FPR [105, 106, 107]. A meta-analysis by Abbasi *et al*. [106] has not detected differences in FPR’s independence of thrombectomy techniques (stent retriever, ADAPT, or combined use [e.g., Solumbra]).

Likewise, FPR is an independent predictor of functional outcomes in patients with posterior circulation stroke caused by an occlusion of the BA or the dominant vertebral artery [108, 109, 110]. Ultimately, independently of the device, technique, or site of the occluded vessel, FPR should be the therapeutic goal in acute ischemic stroke requiring MT.

#### 2.4.2 Stent Retriever Thrombectomy

In traditional stent retriever MT, the stent (attached to a wire) is introduced via a micro-catheter. At the side of the occlusion, the stent is released from the catheter and subsequently self-expands, pushing the thrombus against the wall. As the stent is pulled back into the catheter, the thrombus is removed and retracted. Widely used devices include the Solitaire stent retriever (Medtronic, Dublin, Ireland), the Trevo retriever (Stryker, Kalamazoo, Michigan, USA), EmboTrap (Neuravi, Galway, Ireland), and the pRESET thrombectomy device (phenox GmbH, Bochum, Germany). Newly invented smaller devices such as the Tigertriever 13 (Rapid Medical, Yoqneam, Israel) have shown promising recanalization rates in distal vessel occlusions (e.g., A2, M3, and P2) [111].

Depending on the vascular anatomy and potential underlying diseases, endovascular access to the occlusion site can be challenging. In approximately 10% of patients, reperfusion is not achieved (TICI 0/1) [112]. In one-third of patients, this outcome is due to a failure to reach the targeted occlusion site because of either the supra-aortic vessel anatomy, or cervical (e.g., kinking or coiling of the ICA) or intracerebral vessel tortuosity [112, 113]. Curved or angled intracerebral vessels may lead to stent retriever failure or escape of the blood clot from the stent retriever [114]. Potential alternative strategies include the use of ADAPT in cases of angled intracranial vessels, distal access-guiding/intermediate catheters in observing cervical or intracerebral vascular tortuosity, and a coaxial technique using a small-sized diagnostic catheter over a larger-scale BGC in cases with unfavorable anatomy of the aortic arch or transradial access (e.g., aortic disease, transfemoral access failure) [112, 113, 114].

A transradial approach as a first-line strategy does not appear to differ from transfemoral access in terms of duration, accessibility, and complications, according to large retrospective analyses [115, 116]. Further investigations are needed before this method can be recommended as an alternative first-line access strategy.

#### 2.4.3 ADAPT

ADAPT was developed as an alternative approach to perform embolectomy in acute ischemic stroke. The thrombus is removed via first-pass direct aspiration with a large-bore aspiration catheter (e.g., 5MAX ACE [Penumbra]). Because of their higher aspiration capacity, catheters with larger diameters appear to be more effective [117]. The size of an aspiration catheter enables a stent retriever rescue strategy in cases of futile recanalization. Recently invented devices, such as the MIVI Q aspiration catheter system designed to maximize the lumen size, might serve as promising future tools for distal occlusions [118]. ADAPT has been suggested to be associated with less endothelial damage than the use of stent-retrievers [119]. Whether this effect has clinical significance is unknown.

ADAPT was initially investigated in M1 and intracranial ICA occlusions, in which it has been found to decrease the time to recanalization [120, 121]. It has also been found to be effective and safe in M2 and M3, as well as BA occlusions [122, 123, 124, 125, 126]. The Contact Aspiration versus Stent Retriever for Successful Revascularization (ASTER) trial showed neither superiority nor non-inferiority of ADAPT to stent retriever MT (because the study was underpowered) whereas the Comparison of Direct Aspiration versus Stent Retriever as a First Approach (COMPASS) study did confirm non-inferiority of ADAPT in ICA, M1, and M2 occlusions (mRS 0–2; aspiration: n = 69 [52%], stent retriever: n = 67 [50%]; p [non-inferiority] 0.0014) [122, 127, 128]. The main limitations were a high percentage of rescue therapy in the ADAPT group (ASTER: 32.8%; COMPASS: 21%) and an uneven distribution of the localization of LVO (ASTER: M2 27.6% in direct aspiration versus 17.6% in the stent retriever cohort). A recent meta-analysis suggested higher recanalization rates with ADAPT [129]. However, this finding was mainly driven by observational data and did not interfere with the outcome overall. Leading the way to individualized decision-making, Liao *et al*. [117] have found ADAPT to be superior in embolic vessel occlusion than in occlusions associated with intracranial atherosclerosis.

#### 2.4.4 Proximal Balloon Occlusion

Large-scale balloon guide catheters (BGC) can be placed proximally to the occlusion site. When inflated, they generate blood-flow arrest while retrieving the thrombus. BGC can be used in combination with both stent retriever MT and ADAPT [130, 131, 132]. BGC appear to be associated with improved procedural and functional outcome parameters in observational data [130, 131, 132, 133]. Studies have reported a higher FPR, shorter time to recanalization, and fewer attempts to achieve excellent reperfusion [133, 134]. Anterograde flow arrest during the retrieval of the clot (via direct aspiration or stent retriever) leads to a decrease in distal embolization and embolization to new territories [135, 136]. Therefore, BGC appear to facilitate good functional outcomes [137, 138]. Further developments such as the Walrus BGC are under investigation [139]. Current AHA/ASA guidelines recommend using BGC [35].

#### 2.4.5 Combined Use of Stent Retriever and Distal Aspiration

Combined approaches using a stent retriever and distal aspiration aim to achieve the advantages of each technique. A combination of approved stent retriever devices and large-bore aspiration catheters is used (e.g., Solumbra; Solitaire stent, and ACE [Penumbra]) [140, 141]. The large-bore aspiration catheter is advanced via a microcatheter proximally to the thrombus. The stent retriever device is placed around the thrombus, as performed in stent retriever MT. Under continual aspiration the stent is retracted into the aspiration catheter and removed (together with the aspiration catheter if resistance is felt) [140]. In alternative approaches (e.g., Stent retriever Assisted Vacuum-locked Extraction [SAVE] or Continuous Aspiration Prior to Intracranial Vascular Embolectomy [CAPTIVE]), the thrombus is captured between the catheter tip and stent retriever while both are retracted as a unit (without the stent retriever being introduced into the aspiration catheter) [10, 142, 143]. The Balloon guide with large bore Distal access catheter with Dual Aspiration with Stent-retriever as Standard Approach (BADDASS) and EmboTrap Pinched In Catheter (EPIC) techniques also involve combined retraction of the aspiration catheter and stent retriever, with additional mandatory use of a BGC [144, 145].

Observational data have indicated higher FPR, shorter groin puncture to recanalization times without increased periprocedural complications, and advantages in functional outcomes in patients with occlusion of the intracranial ICA, or the M1 or M2-segment of the MCA [143, 144, 145, 146, 147, 148]. Yet, the ASTER2-trial, published in 2021, has not indicated differences in total or near-total reperfusion (eTICI 2c/3) between a combined approach and stent retriever MT (the use of BGCs in both groups was mandatory) [149]. As noted by the authors, the study was underpowered to detect smaller but potentially relevant differences between groups. In addition, novel technical developments such as very large-bore catheters could further increase aspiration capability [149]. Switching from either stent retriever MT or ADAPT to a combined approach as part of a rescue strategy after futile recanalization might improve recanalization rates [150, 151].

### 2.5 Recanalization

#### 2.5.1 Distal Recanalization in the Anterior Circulation

2.5.1.1 M2 and Beyond

No rationale based on the current literature exists for excluding patients with an M2 occlusion from endovascular therapy. MT for M2 occlusions can achieve similar recanalization rates to those for M1 occlusions, without an increase in symptomatic intracranial hemorrhage [152, 153]. According to data from the HERMES collaboration, patients with M2 stroke benefitted from MT under the respective trial protocols [25]. MRS 0–2 was achieved in 58.2% of patients versus 39.7% in the IVT group (OR 2.39 [1.08–5.28]; *p* = 0.03). Other analyses have confirmed this finding [154, 155]. A recent meta-analysis has found a superior frequency of functional outcomes in M2 than M1 MT [156]. A rate of excellent reperfusion (mTICI 2c/3) of 73.1% has been reported in an Italian registry study [157]. This value is considerably higher than that in patients treated with IVT alone (MT: n = 30 [79%]; IVT plus MT: n = 21 [75%]; IVT alone: n = 24 [44%]; *p* = 0.001) [158]. Preliminary data have not indicated differences between ADAPT and stent retriever MT in terms of recanalization rates [159]. Whether proximal and distal M2 occlusions achieve similar results or require different therapeutic approaches warrants further investigation. Although some authors have shown promising results in patients with M3 occlusion, the question of how far distally one can go remains unanswered [160].

2.5.1.2 Anterior Cerebral Artery

Little information is available on MT for ACA territory stroke. Although limited by sample size (as many as 30 patients), the available data suggest the feasibility and safety of this modality in proximal (A1) and distal (A2, A3) occlusions [160, 161, 162, 163, 164, 165]. Whether ACA occlusions should be treated with MT rather than IVT is a matter of debate: clinical deficits are usually milder than compared to stroke in other territories, and the outcomes are often determined by accompanying MCA infarction or occlusion of the carotid-T [161, 162]. However, even in medium vessel occlusions (including A2 and A3), recanalization occurs in less than half of patients after IVT [76]. Vessel diameters and anatomical findings can make access with large-bore catheters challenging, smaller devices and microcatheters with better access capability for distal vessel occlusions have been developed and investigated (e.g., 3MAX [Penumbra], 5-French SOFIA [MicroVention, California, USA]) [162, 165].

#### 2.5.2 Posterior Circulation

2.5.2.1 Basilar Artery

Basilar artery occlusion is a devastating disease that may potentially evolve into locked-in syndrome or death. Mortality rates as high as 50% are observed despite endovascular therapy or IVT [166]. Outcomes depend not only on rapid recanalization, but also on collateral flow, lower pre-treatment NIHSS score, and stroke localization [167, 168, 169, 170, 171]. An early pontine infarction might decrease the chances of good outcomes [172]. In registry data, MT (plus IVT) has not been found to be superior to IVT alone, although a trend toward a greater improvement in severely affected patients has been observed [173, 174].

The Endovascular treatment versus standard medical treatment for vertebrobasilar artery occlusion study (BEST) study and the Basilar Artery International Cooperation Study (BASICS) have not found a benefit of one approach over the other, although a “substantial benefit of endovascular therapy” could not be excluded [175, 176]. BASICS included patients within 6 hours of symptom onset without an NIHSS score threshold. Favorable functional outcomes (defined by mRS scores of 0–3) occurred in 44.2% of MT patients and 37.7% of the control group (best medical treatment including IVT; risk ratio 1.18 [0.92–1.50]). In moderately affected patients with an NIHSS score 10–19, the absolute risk reduction was 12.2% (mRS score of 0–3: 38.7% versus 26.5%; risk ratio 1.55 [1.06–2.27]) in an underpowered cohort [176]. BEST included BA occlusions within 8 hours of symptom onset. The study was terminated early because of a high crossover rate (22% of patients in the control group (IVT, best medical treatment) received endovascular treatment) [175]. Whereas MT and controls did not differ in terms of the primary endpoint (mRS 0–3: n = 28 [42%] versus n = 21 [32%]; OR 1.74 [0.81–3.74]), MT was found to be superior in the as treated (subgroup) analysis (mRS 0–3: 47% [MT] versus 24% [controls]; OR 3.02 [1.31–7.00]). Both studies had poor recruitment rates, thus potentially indicating that many patients were treated outside the respective trials. Preliminary results of the Endovascular treatment for acute basilar artery occlusion (ATTENTION) trial have been presented at the ESOC 2022 in Lyon, France [177]. Patients with a BA occlusion (within 12 hours of symptom onset), an NIHSS >10, and an age-dependent PC-ASPECTS (above 80: >8 points; below 80: >6 points) were included. No treatment was allowed outside the study at participating centers. The proportion of good functional outcomes (mRS 0–3) was significantly higher in the MT group (46%) than the group receiving the best medical treatment (22.8%; risk ratio 2.1 [1.5–3.0]). Current guidelines strongly advise considering MT for basilar artery occlusion in carefully selected patients [34, 35, 36]. Imaging criteria (e.g., site and expansion of DWI lesions), clinical symptoms, and time from symptom onset (e.g., within the 6-hour time window) might help identify suitable candidates.

2.5.2.2 Posterior Cerebral Artery

No RCT data have been reported on MT for PCA occlusions. In P1 occlusions, MT has been shown to be effective in terms of both recanalization rates and safety [178]. Technical feasibility has also been demonstrated in distal P2 and P3 occlusions [179, 180]. In a meta-analysis published in 2021, MT and IVT have not been found to result in differing outcomes and complications (MT n = 201, IVT n = 64; mRS 0–2 OR 1.5 [0.8–2.5]) [181]. Especially regarding visual deficits and executive functions, MT might decrease persistent disabilities [182, 183]. Both deficits strongly interfere with daily-life activities and are underrepresented in NIHSS and mRS score assessments [184]. Whether the knowledge of specific (and not NIHSS-relevant) deficits might have influenced decision-making in a retrospective patient population is unknown. Focusing on NIHSS and mRS scores alone might underestimate the actual treatment effect in PCA stroke. Until RCT data are published (DISTAL [NCT05029414]), according to our experience, MT is a reasonable therapeutic option for selected patients with proximal PCA occlusion [185].

2.5.2.3 Cerebellar Arteries

Data have been published on MT for cerebellar artery stroke (superior cerebellar artery, posterior inferior cerebellar artery, and anterior inferior cerebellar artery). A retrospective multinational study has identified 16 (out of 668) patients treated with MT of a cerebellar artery, mainly after MT of the BA or the PCA [186]. MT has been found to be feasible with a high rate of periprocedural complications. Whether MT is an alternative to IVT in these patients remains unclear.

### 2.6 Collaterals

Collateral flow is crucial in maintaining the perfusion of the penumbral tissue and is supported by leptomeningeal collaterals and anatomic determinants in the circle of Willis (anterior and posterior communicating artery). Poor collaterals lead to greater infarct volumes and faster progression of the ischemic core [187, 188, 189]. An initially reduced CBV is associated with poor collateral status, indicating further growth of the infarct volume [190].

Several scores have been suggested to grade the collateral status on the basis of CT-A, MRI, or angiographic findings. Tan *et al*. [191] have described a four-point scale based on contrast agent filling distally to the occlusion in CT-A. The score reported by Miteff *et al*. [192] analyzes CT-A images to determine whether vessels can be seen in the Sylvian fissure, cannot be seen at all, or are completely reconstituted distally. More recently, a six-point collateral score (mCTA collateral score; pial arterial filling score) has been described [193]. The mentioned CT-A collateral scores can be seen in Table 4 (Ref. [191, 192, 193]). In MRI, the FLAIR vascular hyperintensity score, based on a FLAIR hyperintense vessel ASPECTS, can be used [194]. The American Society of Interventional and Therapeutic Neuroradiology/Society of Interventional Radiology of the Society of NeuroInterventional Surgery score is based on DSA findings and might be the most precise score correlating well with penumbral tissue and the ischemic core [96, 195]. However, as determined during the intervention, it cannot be used for initial patient selection.

**Table 4.** **Collateral scores, as depicted in CT-A**.

*grade*	Tan *et al*. [191]	Miteff *et al*. [192]	mCTA collateral score [193]
0	Absent collateral supply to the occluded MCA territory	n.a.	No vessels visible in the affected hemisphere in any phase
1	Collateral supply filling ≤50% but >0% of the occluded MCA territory	Contrast opacification seen in only the distal superficial branches	Only a few vessels visible in the affected hemisphere in any phase
2	Collateral supply filling >50% but <100% of the occluded MCA territory	Vessels can be seen at the Sylvian fissure	A filling delay of two phases in the affected hemisphere with significantly fewer vessels in the ischemic territory, or one phase delay showing regions without visible vessels
3	100% collateral supply of the occluded MCA territory	Vessels reconstituted distal to the occlusion	A filling delay of two phases in the affected hemisphere, or a delay of one phase with significantly fewer vessels in the ischemic territory
4	n.a.	n.a.	A filling delay of one phase in the affected hemisphere, but comparable extent and prominence of pial vessels
5	n.a.	n.a.	No filling delay compared with the asymptomatic contralateral hemisphere; normal pial vessels in the affected hemisphere

CT-A, CT angiography; MCA, middle cerebral artery; n.a., not applicable.

In patients with MT, pre-treatment collateral status is associated with the final infarct volume and reperfusion rates [196, 197]. Poor collaterals, according to the Miteff score, are associated with fatal outcomes [198]. In an Italian registry of MT in patients beyond 6 hours after symptom onset, combined collateral assessment and CT-P mismatch has been found to be safe without increasing intracerebral hemorrhage [199]. Although collateral status cannot be the sole parameter in selecting late-presenting patients, it might provide valuable information for predicting functional outcomes [200].

### 2.7 Special Situations

#### 2.7.1 Tandem Occlusions

In approximately 10–20% of patients with anterior circulation LVO, stroke is caused by tandem occlusions [201]. Tandem occlusions are defined as complete or near-total occlusion of the extracranial ICA with an additional intracranial LVO. This definition is somewhat limited, because the causal link between the extra- and intracranial pathology is not highlighted. Beyond an LVO, the extracranial lesion might also trigger an intracranial medium or small vessel occlusion that is not suitable for endovascular therapy but might lead to severe symptoms. Overall, two mechanisms can be causal: local atherosclerotic disease or dissection of the extracranial ICA (discussed below) [202]. Atherosclerotic stroke appears to be more severe. Several treatment approaches have been proposed, and there is no consensus regarding which strategy to choose [203]. An anterograde approach (ICA stenting followed by MT), a retrograde approach (MT followed by ICA stenting), balloon angioplasty (without permanent stent placement) together with MT, and a conservative approach with MT only in the acute stroke setting have been proposed and investigated [201, 204, 205, 206, 207]. A meta-analysis from 2018 has not indicated differences in outcomes among these approaches [206]. Recent data suggest an overall benefit of emergent stenting (retrograde approach) in terms of recanalization and outcomes [201, 207]. Larger RCTs comparing the different endovascular options are currently recruiting participants (e.g., EASI-TOC [NCT04261478]) [208]. Prior IVT (in eligible patients) appears to be safe and to further improve recanalization rates [206, 209, 210]. Bracco *et al*. [211] have recently reported “hemodynamic” tandem occlusion with an acute total or sub-total occlusion of the extracranial ICA without sufficient collateral compensation. Emergent stenting restoring the antegrade blood flow might be crucial [211]. This topic requires further investigation.

The main complication of stent treatment is early stent thrombosis, which has been observed in up to 19% of patients in a retrospective cohort [212]. Most of these patients were treated with a single platelet aggregation inhibitor. This finding might underscore the need to strictly follow a dual platelet aggregation regime even in patients with acute stroke [212]. However, this is associated with an elevated risk of consecutive hemorrhagic complications, because an increase in symptomatic and asymptomatic intracerebral hemorrhage with dual anti-platelet treatment has been reported [213, 214]. Other (retrospective) data have indicated that the risk might be overestimated and that these hemorrhages do not interfere with functional outcomes [215]. Whether hemorrhagic complications differ between a “per protocol” stent placement (as in the treatment of tandem occlusions) and a rescue procedure (indicating a longer treatment duration and multiple thrombectomy passages) cannot be answered.

Data on vertebrobasilar tandem occlusion are scarce [216, 217]. However, this etiology might not be uncommon [216]. Endovascular therapy, including angioplasty or stent placement, might be feasible and safe in selected patients. Further studies are warranted to detect treatment effect complications and identify patients for whom therapy is suitable.

#### 2.7.2 Stenting of Intracranial Atherosclerotic Stenosis during Endovascular Stroke Therapy

In the Stenting versus Aggressive Medical Management for Preventing Recurrent Stroke in Intracranial Stenosis (SAMMPRIS) trial, primary stenting of intracranial stenosis has been found to be inferior to the best medical treatment [218]. In intracranial atherosclerotic LVO, stenting might be used as a rescue therapy (bail-out procedure) in the case of MT failure or early re-occlusion [219, 220]. In both anterior and posterior circulation stroke, permanent stent placement might be a strategy to secure revascularization in an otherwise unfavorable situation, in selected patients only [213, 221]. In a retrospective analysis, 44.8% of patients experienced a good functional outcome after the rescue procedure, whereas the frequency of symptomatic ICH was high (10.5%) [222].

#### 2.7.3 Dissection and Extracranial Bail-Out Stenting

According to current consensus, extracranial ICA dissection should be treated with either oral anticoagulation for 3–6 months or platelet aggregation inhibitors. Information on emergency extracranial ICA stenting due to dissection is limited to smaller observational studies and case reports. Most of the data have focused on endovascular strategies in tandem occlusions due to ICA dissection [223, 224, 225]. In a pooled analysis from registry data, emergent stenting in tandem occlusions has been suggested to be effective and safe, with a slight increase in mainly asymptomatic ICH [223, 224]. Although both the anterograde and the retrograde approaches might be feasible, some authors have suggested a more conservative approach with stent placement only in cases of insufficient collateralization [225, 226, 227].

Even though the outcomes in ICA dissection are favorable, outcomes deteriorate in cases of complete or near-total occlusions caused by ICA dissection [228, 229]. Smaller case series have suggested a beneficial effect of ICA stenting in those patients, with treatment aimed at reperfusion, consolidation of the hemodynamic status, and prevention of (future) emboli [228, 229].

#### 2.7.4 MT in Octo- and Nonagenarians 

Patient-specific characteristics such as age or pre-stroke disability should not exclude patients from endovascular therapy when imaging criteria support MT [230, 231, 232]. Although the overall outcomes in octo- and nonagenarians are poorer than those in patients below 80 years of age, these patients benefit from endovascular stroke therapy [230, 233]. Older patients and those with (unspecified) pre-stroke disability appear to show similar improvement rates to those in independently living patients, and the pre-stroke functional status can be attained [232]. In the case of active cancer, MT can have beneficial effects in selected patients, although the overall mortality is high, owing to the underlying disease [234].

#### 2.7.5 Recurrent LVO

Repeated MT is observed in approximately 1.5–6.6% of patients after initial endovascular therapy [235, 236, 237]. An early re-occlusion at the site of the initial MT must be distinguished from a recurrent LVO affecting the same or any other blood vessel after a period of weeks or months [236, 237, 238, 239, 240]. Risk factors associated with early re-occlusion are atherosclerotic etiology, residual thrombus material, and stenosis after MT [237, 238]. Because these patients might be at risk, prolonged and more intensive post-interventional monitoring should be evaluated [235]. Re-MT is feasible and safe, but the overall outcomes seem to be comparatively poor [237, 238, 240]. Nevertheless, endovascular therapy should not be withheld, because mRS 0–2 is observed in as many as 30–46% of patients in retrospective cohorts [241].

The main cause of a recurrent LVO after initial hospitalization is cardioembolic stroke, which is attributed mainly to a lack of anticoagulation [239, 242]. In retrospective cohorts, functional outcomes or improvements in patients with recurrent MT have been found to be similar to those after first-time MT [238, 239, 242].

#### 2.7.6 Low ASPECTS

RCT data are lacking regarding MT’s beneficial effects (or harms) in patients with large early infarction, defined by CT ASPECTS <6. Several studies are ongoing (e.g., TENSION [NCT03094715], SELECT-2 [NCT03876457], LASTE [NCT03811769], and TESLA [NCT03805308]) [243, 244, 245, 246]. The eligibility criteria differ in time to randomization, baseline NIHSS score, CT/DWI ASPECTS thresholds (between 0–5 and 3–5, depending on age), or imaging modalities (CT-P in SELECT-2), indicating uncertainty in patient selection. Retrospective data suggest a potential benefit of low-ASPECTS MT [247, 248, 249, 250]. This effect appears to be time-dependent [247]. In an early treatment cohort (a median 173 min from last seen well time to admission), the rate of symptomatic intracerebral hemorrhage did not increase [248]. In 2022, The Recovery by Endovascular Salvage for Cerebral Ultra-Acute Embolism–Japan Large Ischemic Core Trial (RESCUE-Japan LIMIT) was published [251]. N = 203 patients with ASPECTS 3-5 (detected on NCCT within 6 hours of symptom onset or MRI [6–24 hours of symptom onset; DWI ASPECTS without demarcation in FLAIR sequences]) were randomized to best medical treatment versus MT. IVT in a dose of 0.6 mg per kilogram body weight was allowed in eligible patients. Functional outcome was superior in the MT group (mRS 0–3; MT: 31%, control group: 12.7%; OR 2.43 [1.35–4.37]). There was an increase in overall intracranial hemorrhage but not in symptomatic intracranial hemorrhage. Generalizability is limited by an entirely Japanese population and a low percentage of patients (in both treatment groups; 28.4% in the medical treatment group, 26.7% in MT) getting IVT. Nevertheless, the study might point towards a further expansion of MT indications. Endovascular treatment in patients presenting with ASPECTS <6 requires individual and thorough decision-making, with a special focus on the duration of symptoms, patient age, and pre-stoke morbidity.

#### 2.7.7 Low NIHSS Score

Patients presenting with a low NIHSS score at admission have been excluded in most MT trials. However, “mild” deficits, such as aphasia, hemianopia, or hemi-ataxia cumulating in an NIHSS score of 0–5, can be disabling and limit functional independence. In a retrospective French cohort study, 12% of patients with LVO and a baseline NIHSS score <5 experienced early neurological deterioration within 24 hours after IVT [252]. Deterioration was associated with poor outcomes. Successful reperfusion in low-NIHSS score cohorts with M1 and M2 occlusions has been found to lead to better short and long-term outcomes [253, 254]. A meta-analysis including data on anterior and posterior circulation stroke has demonstrated the potential superiority of MT to the best medical treatment (n = 581 patients; mRS 0–2: OR 1.68 [1.08–2.61]) [255]. Data indicating a potential benefit in specific patient populations (on the basis of the site of vessel occlusion, collateral status, or penumbral imaging) are lacking. RCTs are currently recruiting participants, such as MOSTE [NCT03796468] and ENDOLOW [NCT04167527] [256, 257].

### 2.8 Procedural Complications

#### 2.8.1 Vessel Dissection and Perforation

Procedural complications during MT occur in up to 15–20% of patients [258, 259]. According to an Italian registry study, the complications include decreasing frequency distal clot embolization (7.6%), symptomatic intracerebral hemorrhage (7.4%), subarachnoid hemorrhage/arterial perforation (2.9%), dissection (1.7%), and access site complications (0.6%) [260].

Vessel perforation is one of the most feared complications and is observed in 0.9–4.9% of patients [18, 19, 20, 21, 22]. The risk increases during the occlusion site maneuver (particularly in situations with access difficulties), passing the occlusion site with a micro-wire or micro-catheter (particularly when observing resistance), and stent or aspiration catheter withdrawal [259, 260, 261]. Atherosclerosis and distal vessel occlusions have been discussed as additional risk factors, whereas MT in later time windows does not appear to increase perforation rates [262]. If a perforation occurs, the perforating device should be kept in situ, because it might at least partially occlude the vessel [258]. Further therapeutic strategies include lowering blood pressure, reversing anticoagulation, and inflating an intracranial balloon at the perforation site (for approximately 5–10 minutes) [258, 259, 260, 261]. In persistent bleeding, the perforated vessel must be sacrificed [258, 263].

A periprocedural dissection is observed in any vessel being manipulated. This complication is observed in 0.6–3.9% of MT cases [18, 19, 20, 21, 22]. Asymptomatic dissection without stenosis or a prominent dissection membrane might be treated with platelet aggregation. In the case of blood flow disturbances, stent placement is necessary [258, 264].

#### 2.8.2 Distal Embolization

Embolization to new or previously non-affected vascular territories is seen in 1–8.6% of cases [18, 19, 20, 21, 22, 258, 259]. It is the most frequent procedural complication in MT. During retrieval of the thrombus parts of the clot can migrate to new vascular territories, or can break up and disseminate into tiny branches downstream, thus affecting parts of the same vascular territory that had previously been spared. Retrieval of a proximal clot increases the risk of embolization [265]. Embolization to new territories is associated with disability and mortality [266, 267]. MT in posterior circulation stroke appears to be associated with higher incidence of both distal embolization and embolization to new territories [268]. Proximal emboli might be removed immediately with the thrombectomy device in use or through aspiration. For distal embolization, intra-arterial thrombolysis might be an option [269]. BGCs appear to considerably decrease the risk of distal embolization [132, 133, 265].

### 2.9 Reperfusion Hemorrhage

Reperfusion hemorrhage is a rare but severe complication after recanalization therapy. Post-interventional hyperperfusion can result in either cerebral edema or intracerebral hemorrhage, and must be distinguished from complications directly associated with the intervention (e.g., bleeding caused by IVT, vessel wall perforation). The most probable underlying mechanism is impaired cerebral autoregulation, particularly in cases of preexisting intra- or extracranial stenosis [270]. Vessel wall injuries and a subsequent increase in permeability observed after MT might be causal, particularly in situations with a sudden increase in blood flow [270]. Clinical features include neurological worsening and coma, headache, and epileptic seizures. Reperfusion hemorrhage is a well-known condition after carotid stenting or carotid endarterectomy [271]. Growing evidence indicates that this condition is common after MT [270, 272, 273]. Shimonaga and colleagues have described (asymptomatic) hyperperfusion in up to 28% of MT patients in a small retrospective cohort [273]. Reperfusion injury might be an important cause of neurological deterioration after MT [251]. A number of clinical and radiological risk factors, such as low baseline ASPECTS, multiple thrombectomy attempts, higher CT-A clot burden, hyperglycemia/diabetes mellitus type 2, prior use of antiplatelet therapy, and elevated mean arterial BP, are discussed [274, 275, 276].

BP lowering <140 mmHg after carotid stenting or carotid endarterectomy can prevent reperfusion hemorrhage [270]. The effect of strict BP targets after MT remains a matter of debate. Depending on revascularization status (TICI 2b/2c/3 versus TICI 1/2a) and BP targets (<160 mmHg, <140 mmHg, or <120 mmHg), retrospective and observational data have shown variable associations of higher BP with disability, deterioration in functional outcome, and symptomatic intracranial hemorrhage [277, 278, 279]. Even short-term increases in BP might lead to poorer outcomes, thus indicating the importance of BP variability [280]. The DAWN protocol investigating MT in late or unknown time windows aimed at a post-interventional BP target <140 mmHg [281]. A large individual patient data meta-analysis published in 2022 has suggested that elevated BP (per 10 mmHg, no threshold mentioned) is associated with intracerebral hemorrhage, early deterioration, mortality, and functional dependence [282].

The BP-TARGET trial has not detected differences in the rate of intracerebral hemorrhage comparing BP goals <130 mmHg and 130–185 mmHg (OR 0.96 [0.60–1.51]) [283]. However, only a minor BP-difference was observed between groups (128 mmHg versus 138 mmHg), the time in the target range for both groups was moderate (61% for the <130 mmHg group versus 66.6%), and BP measurement was non-invasive, thus allowing for undetected BP-variability. The rate of the primary outcome concerning the BP range followed a U-shaped curve with a nadir between 110–140 mmHg [283]. Pending the results of ongoing RCTs (BEST-II [NCT04116112], OPTIMAL-BP [NCT04205305], and ENCHANTED 2 [NCT04140110]), a post-interventional BP-target of <140 mmHg appears reasonable [284, 285, 286].

### 2.10 Long-Term Outcomes of Survivors

RCT follow-up data have shown that the beneficial effects of MT remain stable [287, 288]. In MR CLEAN, 37.1% of patients had good functional outcomes (mRS scores of 0–2) with patient independence 2 years after MT, compared with 23.9% in the IVT-only group (OR 2.21 [1.30–3.73]) [287]. A meta-analysis by McCarthy has concluded that MT leads to good long-term follow-up results in patients, as compared with the 90-day follow-up results [282]. A short delay until initial improvement appears to be a robust indicator of long-term outcomes [289, 290, 291]. This is facilitated by factors such as time to reperfusion and successful recanalization (TICI 2b/2c/3) [292]. Right-hemispheric stroke and high NIHSS scores at discharge might lead to further decline [293]. Other factors such as a systematic inflammation response might be associated with poor outcomes despite successful and rapid recanalization [294]. The understanding of this effect may help identify additional targets to improve outcomes after MT. Nonetheless, 25% of patients not showing early improvement eventually gain functional independence [295].

Beyond functional outcomes, cognitive function is crucial for obtaining independence, self-determination, and quality of life. However, cognitive function is underrepresented in the traditional mRS assessment [296]. MT in an early and late time-window, compared with the standard of care, has been found to improve cognitive function as well as quality of life [296, 297, 298].

## 3. Conclusions

MT in acute ischemic stroke has become the standard of care for patients with anterior circulation LVO. Within an early time-window, MT (plus IVT in eligible patients) is superior to IVT alone. Data on wake-up stroke, posterior circulation stroke, or distal vessel occlusions have led to an expansion of treatment indications. Yet, a number of questions remain unanswered. As part of an individualized decision-making approach, additional subgroups of patient who might potentially benefit from MT must be identified. Therefore, endovascular stroke therapy will remain an exciting and challenging field in the years to come.
